# Risk of Dementia in Patients with Depression or Parkinson's Disease: A Retrospective Cohort Study

**DOI:** 10.1155/2020/8493916

**Published:** 2020-10-10

**Authors:** Ting-Chun Fang, Yu-Hsuan Wu, Yi-Huei Chen, Ching-Heng Lin, Ming-Hong Chang

**Affiliations:** ^1^Department of Neurology, Taichung Veteran General Hospital, Taichung 40705, Taiwan, China; ^2^Department of Medical Education and Research, Taichung Veteran General Hospital, Taichung 40705, Taiwan, China

## Abstract

**Background:**

This study aimed to clarify whether Parkinson's disease (PD) and depression were independent risk factors or with synergic effects in dementia.

**Methods:**

Newly diagnosed PD (*n* = 1213) patients and control subjects (*n* = 4852) were selected from the Taiwan National Health Insurance Research Database from January 2001 through December 2008. Follow-up ended in 2011 with an outcome of dementia occurring or not. This cohort was divided into controls with or without depression, PD only, and PD with depression. The incident rate of dementia and hazard ratio (HR) using Cox's regression analysis were calculated for each group.

**Results:**

When compared with controls without depression as HR 1.00, the adjusted HR for dementia was 3.29 (*p* < 0.001) in the PD only group, 2.77 (*p* < 0.001) in the PD with depression group, and 1.55 (*p*=0.024) in the depression only group. The incident rate of dementia was 29.2 (per 1000 person-years) in the PD only group and 13.2 in the PD with depression group. The effect of PD on dementia in the depression group produced a HR of 0.97 (*p*=0.905).

**Conclusions:**

Parkinson's disease served as a risk factor for dementia. By comparison, depression was not a risk factor for dementia in PD patients, although it did act as a risk factor for dementia.

## 1. Introduction

In addition to the motor symptoms such as bradykinesia, rigidity, tremor, and postural instability, the cumulative risk of dementia in Parkinson's disease (PD) has been confirmed by researchers [1–3]. Depression is also conceived as a good predictor of dementia, and the available evidence indicates shared risk factors and biological changes between dementia and depression [4, 5]. Age, duration, and severity of PD are risk factors of dementia in PD [6], but whether depression and PD demonstrate a synergic effect in the causes of PD dementia (PDD) remains unclear [7]. To answer this query, we have compared the risk of dementia between the general population and PD patients with or without depression.

## 2. Materials and Methods

### 2.1. Study Design and Population

In Taiwan, around 99% of the population participate in National Health Research Insurance (NHRI); hence, the data from the National Health Insurance Research Database (NHIRD) are representative of epidemiology in Taiwan [8]. We randomly selected subjects from Longitudinal Health Insurance Database 2005 who were newly diagnosed with PD from January 2001 to December 2008. The definition and search methodology were based on the International Classification of Diseases, 9^th^ revision, and clinical modification (ICD-9-CM) code 332.0. We took a previous study as a reference for inclusion and exclusion criteria [9]. The subjects were included only with a diagnosis made in three or more consecutive visits at outpatient clinics or with a diagnosis made during hospitalization to increase the validity. The patients who had dementia before PD was diagnosed, had risks for secondary or atypical Parkinsonism, took antidopaminergic medicine more than three times within 3 months before the diagnosis of PD, or never took antiParkinsonian medicine after the diagnosis of PD were excluded. The non-PD control subjects were selected randomly from the database, and they were matched 4 : 1 with PD subjects for age, sex, Charlson comorbidity index (CCI), and the index date (the date of initial diagnosis of the PD subjects). Those who had a diagnosis of dementia before the index date and had a diagnosis of Parkinsonism were excluded from the non-PD control group.

The PD subjects and the non-PD subjects were divided based on depression. Depression was defined based on the ICD-9-CM codes, including 296.2 (major depressive disorder, single episode), 296.3 (major depressive disorder, recurrent episode), 300.4 (dysthymic disorder), 309.0 (adjustment disorder with depressed mood), 309.1 (prolonged depressive reaction), and 311 (depressive disorder, not elsewhere classified). For clinical validation, inclusion was also confined to diagnoses made in three or more consecutive visits at outpatient clinics or made during hospitalization.

We followed up with the subjects from the diagnosis of PD until December 2011. The diagnosis of dementia was the primary clinical outcome. Dementia was diagnosed by ICD-9-CM codes (290, 331.0, 331.2, and A210) in three or more consecutive visits at outpatient clinics or during hospitalization.

The usage and release of the data were approved by the ethics committee of NHRI. Because of anonymous analysis, there was no written consent from participants. The study complied with the Declaration of Helsinki.

### 2.2. Statistical Analysis

Stata version 11.1 was used to perform all statistical analyses. Between PD and non-PD subjects, we used Student's *t*-test and chi-squared test/Fisher's exact test to compared age, gender, Charlson comorbidity index (CCI), depression, hypertension, diabetes mellitus, hyperlipidemia, and ischemic heart disease. The incident rate of dementia was calculated as the number of events divided by person-years. Hazard ratio (HR) was calculated as the risk of dementia between PD and non-PD subjects using Cox's regression analysis. HRs in different groups (depression or not), genders, and ages (<50, 50–64, and ≥65 years old) were calculated to elucidate the impact of PD in each group. Based on previous studies [9–13], adjusted HRs were acquired for age, gender, hypertension, diabetes mellitus, hyperlipidemia, and ischemic heart disease because metabolic syndrome is broadly considered as having an association with PD, depression, and dementia. *p* values< 0.05 and confidence intervals (CIs) for HRs, which excluded value 1.00, were statistically significant.

## 3. Results

### 3.1. Cohort Characteristics

Table 1 shows the characteristics of the study population. Six thousand and sixty-five subjects were included in the study, 1213 of whom were newly diagnosed PD subjects. The prevalence of depression in all of the patients was 11.3%. The occurrence of depression was significantly higher in the PD group than in the non-PD group (27.4% vs. 7.3%, *p* < 0.001). Significantly, more patients had hypertension in the PD group than in the non-PD group (37.3% vs. 33.5%, *p*=0.013). Between the two groups, there were no significant differences in age, gender, Charlson comorbidity index (CCI), or the presence of diabetes mellitus, hyperlipidemia, or ischemic heart disease.

### 3.2. Hazard Ratios of Different Variables for Dementia

In Table 2, the adjusted HR for dementia was 1.10 (95% CI 1.09–1.11, *p* < 0.001) with aging, 1.64 (95% CI 1.30–2.07, *p* < 0.001) in subjects with diabetes mellitus, 0.70 (95% CI 0.52–0.93, *p*=0.014) in subjects with hyperlipidemia, and 1.23 (95% CI 1.00–1.51, *p*=0.047) in subjects with hypertension. In terms of genders and ischemic heart disease, no statistically significant differences were detected for the HRs of dementia. When comparing to the subjects who had neither PD nor depression, the adjusted HRs for dementia were 1.55 (95% CI 1.06–2.25, *p*=0.024) in the PD only group, 3.29 (95% CI 2.66–4.06, *p* < 0.001) in the depression only group, and 2.77 (95% CI 1.88–4.08, *p* < 0.001) in the group with both PD and depression.

### 3.3. Hazard Ratios for Dementia with PD in Different Subgroups

Table 3 reveals a higher incidence of dementia in PD than in the control (24.2 vs. 8.3/1000 person-years). The crude hazard ratio (HR) for dementia in PD was 2.91 (95% CI 2.39–3.53, *p* < 0.001), and the adjusted HR was 3.05 (95% CI 2.51–3.70, *p* < 0.001). In terms of gender, hazard ratios were 2.92 (95% CI 2.19–3.89, *p* < 0.001) and 3.20 (95% CI 2.45–4.17, *p* < 0.001) in female and male groups, respectively. In groups divided by ages, all the groups had a HR greater than 1.00 for dementia in PD. The highest incident rate of dementia was in PD subjects older than 65 years (42.3/1000 person-years). However, the adjusted HR of dementia was highest in PD subjects younger than 50 years (adjusted HR 12.10, 95% CI 2.15–68.04, *p*=0.005) with the lowest incident rate (2.5/1000 person-years). In the subgroup without depression, the adjusted HR for dementia with PD was 3.30, *p* < 0.001. In comparison, the subgroup with depression had crude HR 0.97 (95% CI 0.58–1.61, *p*=0.905) and adjusted HR 1.77 (95% CI 1.06–2.96, *p*=0.03).

Table 4 reports that, without depression, the adjusted HR for dementia with PD was 10.08 (95% CI 4.67–21.76, *p* < 0.001) in the younger group and was 2.99 (95% CI 2.39–3.74, *p* value < 0.001) in the older group. In contrast, HRs did not reach statistical significance in each group with depression (adjusted HR 7.06; *p*=0.074 in the younger group and 1.55; *p*=0.12 in the older group).

## 4. Discussion

A higher prevalence of depression was observed in PD patients in our study which is similar to the findings of other studies [7, 10]. However, the incident rate of dementia in the PD without depression group was higher than in the PD plus depression group. This finding implies that depression might not be a risk factor in dementia of PD. This supports the result of a previous cohort study, where the authors concluded a later age of onset of PD, increased disease severity of PD, and hallucination were predictive factors for dementia development, but depression was not [14]. In contrast, some studies have suggested that depression can precede dementia in PD patients and might be a risk factor for dementia in PD [6, 15]. These opposite conclusions might be caused by different study designs or the difficulty in determining the effect of depression upon dementia in PD due to shared symptoms, such as apathy, psychomotor problems, and sleep disturbances, between depression and dementia. In our study, having depression was associated with a higher rate of dementia; in the discussion about the incidence of dementia with or without PD in the groups having depression, having PD did not make much of a difference in the incidence rate of dementia in this study. This revealed that depression plays an important role in dementia. Previous studies had also revealed depression to be a risk factor of dementia [4, 16]. The specific types of dementia associated with depression continue to be debated. It has been proposed that depression might precede the onset of Alzheimer's disease or vascular dementia [17, 18].

We also divided the PD subjects by age, and it reveals that having PD does increase the risk of subsequent dementia, irrespective of early-onset PD or older-onset PD. However, different characteristics of incidence rates and HRs of dementia were found between these groups. A higher incidence rate detected was in the older-onset group, but HRs were higher in the early-onset group. This finding is consistent with the different progression of pathology between early-onset PD and older-onset PD proposed by others. Older-onset PD is associated with shorter survival and a complex disease course. More rapid cognitive decline is also found in this phenotype associated with higher Lewy-related pathology and additional neuropathologies, such as Alzheimer's disease [19].

There are some limitations to this cohort. First, the data were collected from the Taiwan National Health Insurance Research Database. Therefore, the validity of the diagnosis might be questioned. In a Taiwanese study, the authors randomly selected patients who coded PD, and these patients were examined by experienced neurologists. The sensitivity, specificity, positive predictive value, and negative predictive value were 97.6%, 92.3%, 98.8%, and 85.7%, respectively [20]. To increase the validity, we also set strict selection criteria and enrolled a sufficient sample size for the study. Second, we did not subtype depression according to severity, symptoms, or onset time. Some researchers have supposed that differences in the onset time of depression might have different risks for dementia [21, 22]. Third, we did not subtype dementia in the general population. Furthermore, additional studies about the biological network of PD, depression, and dementia are necessary to identify the relationships and causalities among these conditions.

## 5. Conclusion

PD and depression both serve as important risk factors for dementia. However, depression was not a risk factor for dementia in PD, despite the high prevalence of depression in PD.

## Figures and Tables

**Table 1:** Clinical characteristics of study subjects.

Clinical characteristics	Total	Without PD	With PD	*p*
	(*n* = 6065)	(*n* = 4852)	(*n* = 1213)	
Age, years (mean ± SD)	62.8 ± 17.1	62.8 ± 17.1	62.9 ± 17.0	0.823
Gender				1.000
Female	2925 (48.2)	2340 (48.2)	585 (48.2)	
Male	3140 (51.8)	2512 (51.8)	628 (51.8)	
CCI				1.000
0	3405 (56.1)	2724 (56.1)	681 (56.1)	
1-2	2055 (33.9)	1644 (33.9)	411 (33.9)	
≧3	605 (10.0)	484 (10.0)	121 (10.0)	
Depression	688 (11.3)	356 (7.3)	332 (27.4)	<0.001
Hypertension	2077 (34.2)	1625 (33.5)	452 (37.3)	0.013
Diabetes mellitus	915 (15.1)	713 (14.7)	202 (16.7)	0.088
Hyperlipidemia	807 (13.3)	633 (13.0)	174 (14.3)	0.234
Ischemic heart disease	800 (13.2)	621 (12.8)	179 (14.8)	0.072

*Note*. PD = Parkinson's disease, *n* = case number, SD = standard deviation, CCI = Charlson comorbidity index, and *p* = *p* value. Categorical data were presented as number (%); continuous data were expressed as mean ± standard deviation.

**Table 2:** Hazard ratio for dementia with different variables.

Variable	Adjusted HR^a^ (95% CI)	*p*
Age, years	1.10 (1.09–1.11)	<0.001
Gender		
Female	1.00	
Male	0.98 (0.81–1.19)	0.839
Hypertension	1.23 (1.00–1.51)	0.047
Diabetes mellitus	1.64 (1.30–2.07)	<0.001
Hyperlipidemia	0.70 (0.52–0.93)	0.014
Ischemic heart disease	0.95 (0.74–1.22)	0.693
PD/depression		
Without PD/without depression	1.00	
Without PD/with depression	1.55 (1.06–2.25)	0.024
With PD/without depression	3.29 (2.66–4.06)	<0.001
With PD/with depression	2.77 (1.88–4.08)	<0.001

*Note*. PD = Parkinson's disease, HR = hazard ratio, % = percentage, CI = confidence interval, and *p* = *p* value. ^a^Adjusted HR: adjusted for age, gender, hypertension, diabetes mellitus, hyperlipidemia, and ischemic heart disease.

**Table 3:** Incident rate and hazard ratio for dementia with PD.

Group	Without PD	With PD	Crude HR (95% CI)	*p*	Adjusted HR^b^ (95% CI)	*p*
	Incident rate^a^	Incident rate^a^				
Overall	8.3	24.2	2.91 (2.39–3.53)	<0.001	3.05 (2.51–3.70)	<0.001
Female	7.9	22.5	2.87 (2.16–3.82)	<0.001	2.92 (2.19–3.89)	<0.001
Male	8.7	25.8	2.94 (2.26–3.83)	<0.001	3.20 (2.45–4.17)	<0.001
Age, years						
<50	0.2	2.5	10.14 (1.97–52.26)	0.006	12.10 (2.15–68.04)	0.005
50–64	1.3	13.1	9.84 (4.51–21.49)	<0.001	9.59 (4.38–20.98)	<0.001
≧65	15.4	42.3	2.78 (2.26–3.42)	<0.001	2.74 (2.22–3.36)	<0.001
Depression						
No^c^	7.9	29.2	3.71 (3.00–4.58)	<0.001	3.30 (2.67–4.07)	<0.001
Yes^c^	13.7	13.2	0.97 (0.58–1.61)	0.905	1.77 (1.06–2.96)	0.030

*Note.* PD = Parkinson's disease, HR = hazard ratio, CI = confidence interval, *n* = case number, % = percentage, and *p* = *p* value. ^a^Per 1000 person-years. ^b^Adjusted HR: adjusted for age, gender, hypertension, diabetes mellitus, hyperlipidemia, and ischemic heart disease. ^c^“No” = without depression; “yes” = with depression.

**Table 4:** Incident rate and hazard ratio for dementia with PD in depression subgroups with cutoff age at 65.

Group	Without PD	With PD	Crude HR (95% CI)	*p*	Adjusted HR^b^ (95% CI)	*p*
	Incident rate^a^	Incident rate^a^				
No depression						
<65	0.7	9.1	12.56 (5.84–27.00)	<0.001	10.08 (4.67–21.76)	<0.001
≧65	14.8	44.8	3.10 (2.48–3.88)	<0.001	2.99 (2.39–3.74)	<0.001

Depression						
<65	1.1	4.6	4.29 (0.53–34.88)	0.173	7.06 (0.83–60.24)	0.074
≧65	22.8	32.6	1.43 (0.82–2.47)	0.206	1.55 (0.89–2.71)	0.120

*Note.* PD = Parkinson's disease, HR = hazard ratio, CI = confidence interval, *n* = case number, % = percentage, and *p* = *p* value. ^a^Per 1000 person-years. ^b^Adjusted HR: adjusted for age, gender, hypertension, diabetes mellitus, hyperlipidemia, and ischemic heart disease.

## Data Availability

The data are available from Taiwan's National Health Insurance Research Database.

## References

[1] Buter T. C., van den Hout A., Matthews F. E., Larsen J. P., Brayne C., Aarsland D. (2008). Dementia and survival in Parkinson disease: a 12-year population study. *Neurology*.

[2] Hely M. A., Reid W. G. J., Adena M. A., Halliday G. M., Morris J. G. L. (2008). The Sydney multicenter study of Parkinson’s disease: the inevitability of dementia at 20 years. *Movement Disorders*.

[3] Perez F., Helmer C., Foubert-Samier A., Auriacombe S., Dartigues J.-F., Tison F. (2012). Risk of dementia in an elderly population of Parkinson’s disease patients: a 15-year population-based study. *Alzheimer’s & Dementia*.

[4] Bennett S., Thomas A. J. (2014). Depression and dementia: cause, consequence or coincidence?. *Maturitas*.

[5] Enache D., Winblad B., Aarsland D. (2011). Depression in dementia. *Current Opinion in Psychiatry*.

[6] Lieberman A. (2006). Are dementia and depression in Parkinson’s disease related?. *Journal of the Neurological Sciences*.

[7] Lieberman A. (2006). Depression in Parkinson’s disease—a review. *Acta Neurologica Scandinavica*.

[8] Wu T.-Y., Majeed A., Kuo K. N. (2010). An overview of the healthcare system in Taiwan. *London Journal of Primary Care*.

[9] Wu Y.-H., Lee W.-J., Chen Y.-H., Chang M.-H., Lin C.-H. (2016). Premotor symptoms as predictors of outcome in Parkinsons disease: a case-control study. *PLoS One*.

[10] Hsu Y.-T., Liao C.-C., Chang S.-N. (2015). Increased risk of depression in patients with Parkinson disease: a nationwide cohort study. *The American Journal of Geriatric Psychiatry*.

[11] Deckers K., van Boxtel M. P. J., Schiepers O. J. G. (2015). Target risk factors for dementia prevention: a systematic review and Delphi consensus study on the evidence from observational studies. *International Journal of Geriatric Psychiatry*.

[12] Yaffe K., Boustani M. (2014). Benzodiazepines and risk of Alzheimer’s disease. *BMJ*.

[13] Koponen H., Jokelainen J., Keinänen-Kiukaanniemi S., Kumpusalo E., Vanhala M. (2008). Metabolic syndrome predisposes to depressive symptoms. *The Journal of Clinical Psychiatry*.

[14] Hobson P., Meara J. (2004). Risk and incidence of dementia in a cohort of older subjects with Parkinson’s disease in the United Kingdom. *Movement Disorders*.

[15] Giladi N., Treves T. A., Paleacu D. (2000). Risk factors for dementia, depression and psychosis in long-standing Parkinson’s disease. *Journal of Neural Transmission*.

[16] Mirza S. S., Wolters F. J., Swanson S. A. (2016). 10-year trajectories of depressive symptoms and risk of dementia: a population-based study. *The Lancet Psychiatry*.

[17] Holmquist S., Nordström A., Nordström P. (2020). The association of depression with subsequent dementia diagnosis: a Swedish nationwide cohort study from 1964 to 2016. *PLoS Medicine*.

[18] Kessing L. V. (2012). Depression and the risk for dementia. *Current Opinion in Psychiatry*.

[19] Halliday G. M., Holton J. L., Revesz T., Dickson D. W. (2011). Neuropathology underlying clinical variability in patients with synucleinopathies. *Acta Neuropathologica*.

[20] Liu C.-C., Li C.-Y., Lee P.-C., Sun Y. (2016). Variations in incidence and prevalence of Parkinson’s disease in Taiwan: a population-based nationwide study. *Parkinson’s Disease*.

[21] Wang L. Y., Shofer J. B., Thompson M. L. (2011). Temporal relationship between depression and dementia. *Archives of General Psychiatry*.

[22] Brommelhoff J. A., Gatz M., Johansson B., McArdle J. J., Fratiglioni L., Pedersen N. L. (2009). Depression as a risk factor or prodromal feature for dementia? Findings in a population-based sample of Swedish twins. *Psychology and Aging*.

